# Endocannabinoids and Endovanilloids: A Possible Balance in the Regulation of the Testicular GnRH Signalling

**DOI:** 10.1155/2013/904748

**Published:** 2013-08-29

**Authors:** Rosanna Chianese, Vincenza Ciaramella, Donatella Scarpa, Silvia Fasano, Riccardo Pierantoni, Rosaria Meccariello

**Affiliations:** ^1^Dipartimento di Medicina Sperimentale Sezione “F. Bottazzi,” Seconda Università di Napoli, Via Costantinopoli 16, 80138 Napoli, Italy; ^2^Dipartimento di Scienze Motorie e del Benessere, Università di Napoli Parthenope, Via Medina 40, 80133 Napoli, Italy

## Abstract

Reproductive functions are regulated both at central (brain) and gonadal levels. In this respect, the endocannabinoid system (eCS) has a very influential role. Interestingly, the characterization of eCS has taken many advantages from the usage of animal models different from mammals. Therefore, this review is oriented to summarize the main pieces of evidence regarding eCS coming from the anuran amphibian *Rana esculenta*, with particular interest to the morphofunctional relationship between eCS and gonadotropin releasing hormone (GnRH). Furthermore, a novel role for endovanilloids in the regulation of a testicular GnRH system will be also discussed.

## 1. Introduction

Endocannabinoids (eCBs)—such as anandamide (AEA) and 2-arachidonoylglycerol (2-AG)—are lipophilic molecules that work as integral part of the endocannabinoid system (eCS), mimicking several actions of Δ^9^-tetrahydrocannabinol (THC), the active principle of *Cannabis sativa*. Although the existence of an intracellular receptor has been suspected, eCBs exert their actions by binding to specific membrane receptors, CB1 and CB2 [1, 2], whose expression is widespread in all species analyzed to date [3]. Unlike 2-AG, AEA also binds to the intracellular site of the type-1 vanilloid receptor (TRPV1), a cation channel receptor also activated by the pungent compound of hot chili pepper, and capsaicin (CAP, 8-methyl-*N*-vanillyl-6-nonenamide) [4]. Other receptors such as GPR55 and GPR119 have been considered putative cannabinoid receptors, however with some hesitation [5].

Although eCBs are lipidic compounds able to traverse plasma membrane by passive diffusion, the existence of a hypothetical eCB transporter has been suggested. In this respect, AEA intracellular carriers belonging to fatty acid binding proteins (FABP) family have been discovered [8]. In addition, eCBs can be also inactivated by a mechanism of cellular reuptake followed by an intracellular degradation mediated by fatty acid amide hydrolase (FAAH) [9] and monoacylglycerol lipase (MAGL) [10]. In neurons, a cytosolic variant of FAAH-1, termed FLAT—which lacks amidase activity but binds AEA with low micromolar affinity—has been considered as an AEA transporter [11]. Endocannabinoid system also includes several enzymes responsible for endocannabinoid biosynthesis such as *N*-acylphosphatidylethanolamine- (NAPE-)specific phospholipase-D (PLD) for AEA [12] and sn-1-diacylglycerol lipase (DAGL) for 2-AG [13].

During the course of the years, the eCS has been characterized and studied from a functional point of view in many species [14–17]. In this regard, the use of nonmammalian animal models has contributed to a better comprehension about the eCS actions, especially in several reproductive events [16, 18–20]. In fact, nonmammalian vertebrates offer a broad spectrum of potentialities, besides, to allow evolutionary speculations. Most of them are seasonal breeders; therefore temperature and photoperiod—easily adjustable in laboratory—deeply control their gonadal activity. In addition, both brain and gonad architecture show morphological features simpler than mammals thus to easily study relationships between different neuroendocrine/paracrine systems [21].

## 2. *Rana esculenta*: An Experimental Model to Study the eCS at Both Central and Testicular Levels

The choice of an appropriate animal model is a basic step in the configuration of an experimental approach. Very often the difficulties found in the determination of molecular mechanisms on the basis of important physiological functions—when studied in mammals—incite to select other animal models, especially nonmammalian vertebrates. With this in mind, the anuran amphibian *Rana esculenta *has been a suitable model for the comprehension of endocannabinoid role in reproduction at both central and testicular levels.

During the annual cycle of this seasonal breeder, the gonadotropin-releasing hormone (GnRH)—the main regulator of gonadal activity—accumulates in the brain in the postreproductive period and is slowly released during the winter stasis to sustain the gonadotropin discharge in order to assess the beginning of a new reproductive wave [22–24]. Furthermore, this amphibian shows a laminated type brain—an archetype of those more elaborated of the higher vertebrates—in which GnRH secreting neurons occupy well-known and distinct areas, differently from mammals in which they are quite scattered in the brain [25]. Additionally, frog spermatogenesis proceeds slowly, orchestrated by environmental factors, testicular mediators, and hormonal milieu characterized by cyclic fluctuations. In particular, in specific periods of the annual sexual cycle it is possible to identify in testis a defined and well-known population of germ cells thanks to a very peculiar cystic organization. This consists in Sertoli cells enveloping clusters of germ cells at a synchronous stage [26, 27].

The characterization of eCS in *R. esculenta* begun in 2006 with the molecular cloning and the expression analysis of *cb1* [28, 29]. As indicated above, endocannabinoid activity requires multiple receptors, and this issue is stressed by the discovery of duplicated genes in fish [30, 31], by the detection of several cannabinoid receptor splicing forms [32–34] as well as by the discussed existence of receptors other than CB1/CB2 [5]. In frog, the characterization of *cb1* did not revealed any splicing form but nucleotide differences among brain/testis cDNA and genomic sequences together with the corresponding amino acidic variations [18, 19, 29] as a consequence of a possible editing process. Such a phenomenon seems to occur in other vertebrates and to affect RNA folding, stability and turnover. However, at present, synonymous and nonsynonymous mutations in *cb1/cb2* and *Faah* genes have been reported in humans and have been linked to several diseases such as metabolic and reproductive disorders, feeding behaviour, obesity, and schizophrenia [35–40].

In amphibian brain, CB1 is widely distributed in the forebrain [41, 42], the encephalic area mainly involved in the control of reproductive functions, being primarily responsible for the biosynthesis of GnRH [21]. As deeply described in the next paragraph, functional crosstalk between eCBs and GnRH system emerged in frog. 

**Figure 1:**
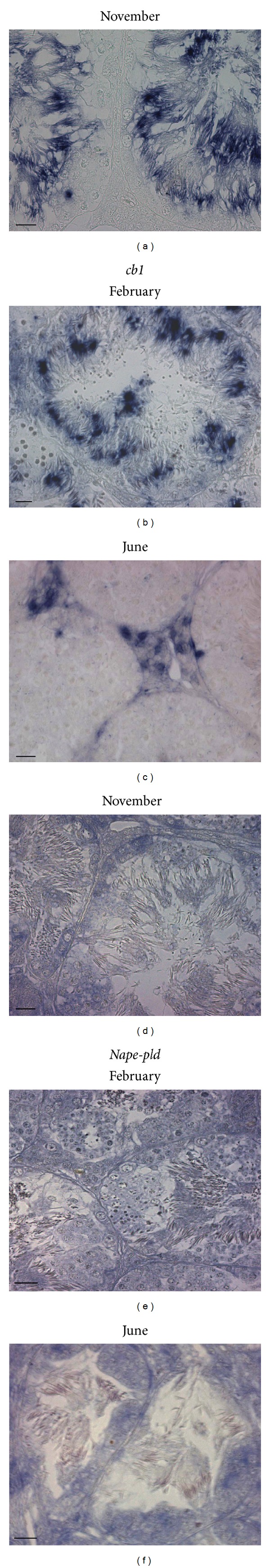
Localization of *cb1* and *Nape-pld* mRNA in the frog testis evaluated by *in situ* hybridization in November (a) and (d), February (b) and (e), and June (c) and (f). Scale bar: 20 *μ*m.

As in other vertebrates and in the central nervous system, *cb1* is widely expressed in frog tissues, gonads included [28]. Fluctuations of *cb1* expression have been reported in both testis and brain during the annual sexual cycle [28] with testicular CB1 mRNA/protein [6, 7, 28] detected in parallel to FAAH in germ cells, especially in elongated spermatids and spermatozoa as observed in other vertebrates (Figures 1(a) and 1(b)) [6, 7, 42–49] and in sea urchin as well [50]. 

In rodents and in germ cells, CB1 has also been detected in Leydig cells suggesting its possible involvement in Leydig cell ontogenesis and steroidogenetic activity [51–53]; interestingly, in frog *cb1* mRNA was only observed in interstitial compartment (Figures 1(b) and 1(c)), and its expression profile well correlates with seasonal testosterone production [54]. Together with the ability to degrade AEA, frog testis might be able to produce eCBs during the annual reproductive cycle as suggested by *Nape-pld* expression and localization [6]. In the germinal compartment *Nape-pld *mRNA has been observed in secondary spermatogonia and spermatocytes cysts as well as in Sertoli cells surrounding primary spermatogonia; the strongest signal has been found in the interstitium throughout the annual sexual cycle (Figures 1(d)–1(f)).

Taken all together, data in frog clearly confirm a deep evolutionarily conserved involvement of eCBs in germ cell progression and sperm cell functions [43–49, 55–57]. Accordingly, as in human, boar, bull, rodents, and sea urchin, also in frog AEA modulates sperm motility [7, 43, 49, 50, 58, 59], indicating an evolutionarily conserved role in the regulation of such a reproductive function.

## 3. Relationship between eCS and GnRH System

The presence of *cb1* in frog brain, mainly in the forebrain and midbrain—as also observed from fish to mammals [14, 41, 60, 61]—has suggested that eCS is able to control reproductive functions through a central regulation. This is in line with the discovery that hypothalamic immortalized GnRH secreting neurons possess a complete eCS, CB1 included [62] and that AEA inhibits GnRH release from rat mediobasal hypothalamus [63]. During the annual sexual cycle, *cb1* mRNA fluctuations are opposite as compared to *GnRH-1* [19, 42]; in particular, in frog diencephalons—the encephalic area mainly involved in the release of GnRH—*cb1* expression shows a peak in December, when low levels of GnRH have been detected [22, 24, 64]. The total CB1 protein content has also been assayed in frog forebrain, midbrain, and hindbrain [16, 19] during the year; intriguingly, GnRH release correlates with the minimal levels of CB1 detected in both telencephalon and diencephalon. Accordingly, neuroanatomical and functional relationships between CB1 and GnRH have been discovered in *R. esculenta* brain by immunofluorescence; in particular, CB1 has been found in a subpopulation of the septal and preoptic GnRH-1 neurons [42]. In addition, the *in vitro* treatment of frog diencephalons with AEA has an inhibitory effect upon *GnRH-1* expression, *via cb1* activation [42]. Such a functional crosstalk between the eCS and GnRH is really more complicated, due to the existence of multiple GnRH and gonadotropin-releasing hormone receptor (GnRH-R) molecular forms in *R. esculenta*. In particular, in frog diencephalons, AEA, with a fine CB1-dependent regulation, is able to decrease *GnRH-1* and *GnRH-2* and increase *GnRH-R1* and *GnRH-R2* expression, with no effect upon *GnRH-R3* [65].

In the last years an emerging idea is that the inhibitory action of eCBs on reproductive functions, especially on GnRH neurons activity, might be pondered by new molecules positively affecting reproduction, such as the kisspeptins [66]. Interestingly, the kisspeptin receptor, *GPR54*, has been cloned and characterized in frog [67], and AEA, *in vivo*, inhibits the hypothalamic GnRH system *via* GPR54 [Chianese et al., unpublished results].

**Figure 2:**
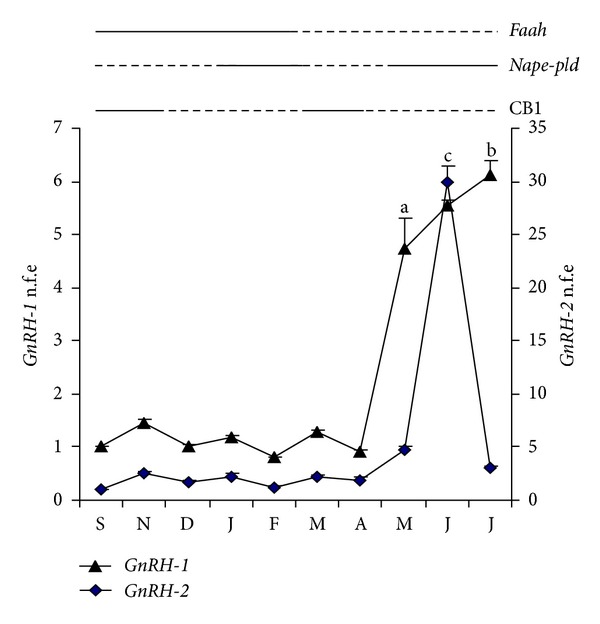
Analysis of *GnRH1*, *GnRH-2*, and some molecular components of the endocannabinoid system in frog testis during the annual sexual cycle. For *GnRH-1*, *GnRH-2*, and *Nape-pld* mRNA data from [6]; for FAAH and CB1 protein data from [7]. Dotted lines: low levels; black lines: high levels. n.f.e.= normalized fold expression. Different letters indicate statistically significant differences.

In the wake of brain analysis, a deep characterization of GnRH system in relation to eCS has been carried out in frog testis as well [6] (Figure 2). CB1 protein peaks have been observed in periods of the cycle characterized by massive formations of postmeiotic cells (September) and during the breeding season (March) with CB1 mainly localized in postmeiotic stages. Interestingly, the expression profiles of testicular GnRHs clearly indicate their increase in postreproductive period, with *GnRH-1* increased expression occurring from May to July and *GnRH-2* expression presenting a single expression spike in June [6]. Thus, in a period in which both CB1 and FAAH proteins are scantly expresseds GnRH is overexpressed (Figure 2). 

**Figure 3:**
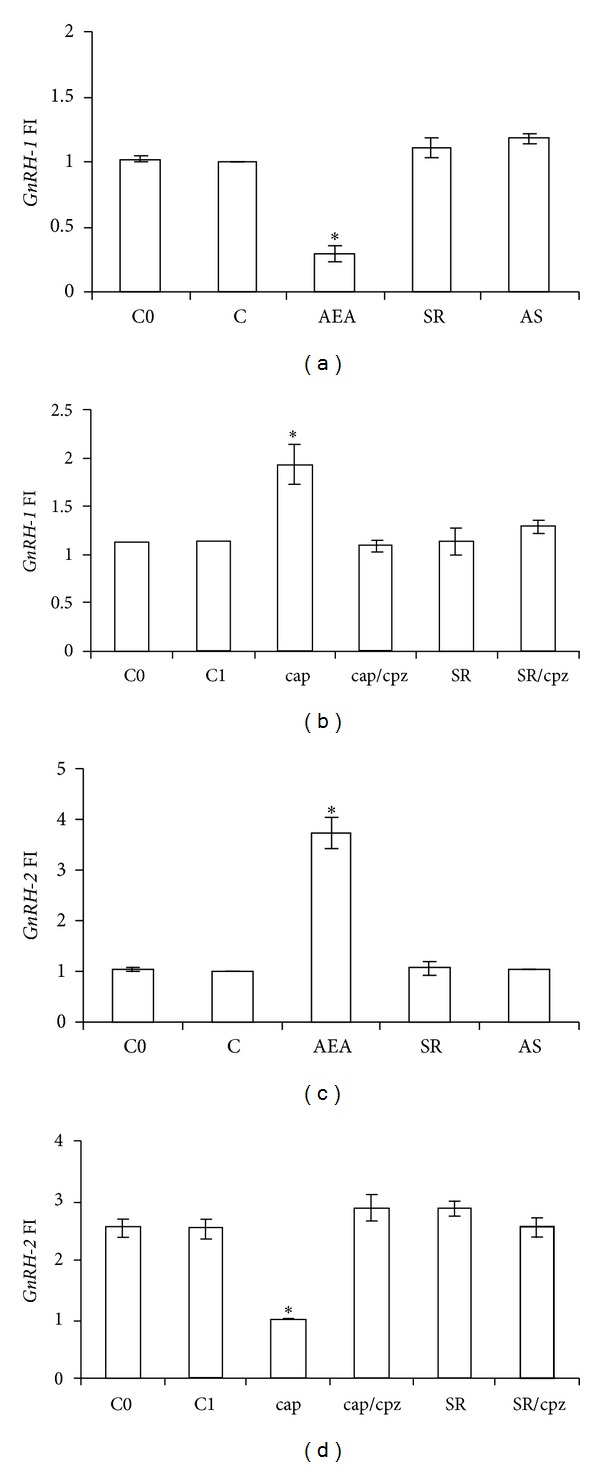
Effects of AEA treatment on *GnRH-1* (a) and *GnRH-2* (c) expression in frog testis collected from June animals (*N* = 5/group) after 1 h of incubation. Incubations have been carried out with AEA 10^−9^ M, SR 10^−8^ M, or both. C0: untreated testis of June; C: control group, testis treated with Krebs-Ringer buffer. Effects of cap treatment on *GnRH-1* (b) and *GnRH-2* (d) expressions in frog testis of June after 1 h of incubation. Incubations have been carried out with cap 10^−6^ M, cpz 10^−5^ M, SR 10^−8^ M, or combinations of cap/cpz and SR/cpz. C0: untreated testis of June; C1: control group, testis treated with Krebs-Ringer buffer. The data in graph are the results of RT-PCR analysis; they are reported as fold increase (FI) calculated comparing the expression of *GnRH-1*/*GnRH-2* to the housekeeping *fp1 *and are representative of three separate experiments at least (*N* = 6). Asterisks indicate statistically significant differences.

**Figure 4:**
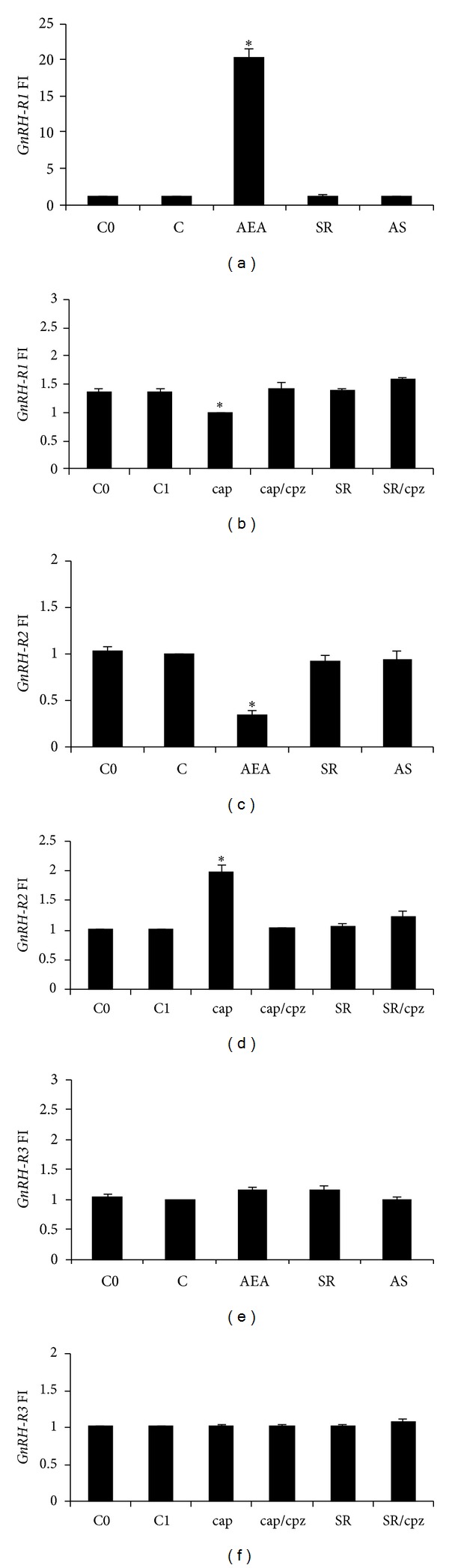
Effects of AEA treatment on *GnRH-R1* (a), *GnRH-R2* (c), and *GnRH-R3* (e) expressions in frog testis collected from June animals (*N* = 5/group) after 1 h of incubation. Incubations have been carried out with AEA 10^−9^ M, SR 10^−8^ M, or both. C0: untreated testis of June; C: control group, testis treated with Krebs-Ringer buffer. Effects of cap treatment on *GnRH-R1* (b), *GnRH-R2* (d), and *GnRH-R3* (f) expressions in frog testis of June after 1 h of incubation. Incubations have been carried out with cap 10^−6^ M, cpz 10^−5^ M, SR 10^−8^ M, or combinations of cap/cpz and SR/cpz. C0: untreated testis of June; C1: control group, testis treated with Krebs-Ringer buffer. The data in graph are the results of RT-PCR analysis; they are reported as fold increase (FI) calculated comparing the expression of *GnRH-Rs *to the housekeeping *fp1 *and are representative of three separate experiments at least (*N* = 6). Asterisks indicate statistically significant differences.

GnRH works as a testicular bioregulator affecting spermatogenesis, sperm release, and fertilization [21, 68, 69], processes also driven by eCBs. With this in mind, we carried out *in vitro* incubations of frog testis with AEA choosing two periods of the annual cycle: June (postreproductive period), when testis is reach in meiotic stages; February (end of the winter stasis), when the upsurge of a new spermatogenetic wave occurs. Intriguingly, frog testis shows a quite different modulation of the GnRH system by AEA in comparison to brain. In fact, in frog diencephalon GnRH-1 and GnRH-2—both hypophysiotropic factors [21]—are localized in the anterior preoptic area, and their transcripts are both inhibited by AEA, whereas in testis they are differently expressed, probably working in different reproductive events. In particular, in June, when spermatogenesis slightly proceeds, an opposite regulation by AEA has been observed since AEA decreases *GnRH-1* and increases *GnRH-2* expression (Figures 3(a) and 3(c)), through *cb1* activation (Figure 4(a)) [6]. Furthermore, a specific modulation by AEA has also been observed on *GnRH-Rs* expression, since AEA upregulates *GnRH-R1* and decreases *GnRH-R2* expression, without any effect upon *GnRH-R3* (Figures 4(a), 4(c), and 4(e)). Interestingly, in February, when testis simply contains quiescent spermatogonia and spermatozoa attached to Sertoli cells, AEA affects *GnRH-2* and *GnRH-R2*, a system supposed to be involved in Sertoli-spermatozoa communication, and does not modulate *GnRH-1/GnRH-R1*, a system supposed to be involved in germ cell progression [6]. Therefore, AEA might modulate testicular GnRH signalling at multiple levels and in a stage dependent manner [6].

## 4. Relationship between Endovanilloids and GnRH System

**Figure 5:**
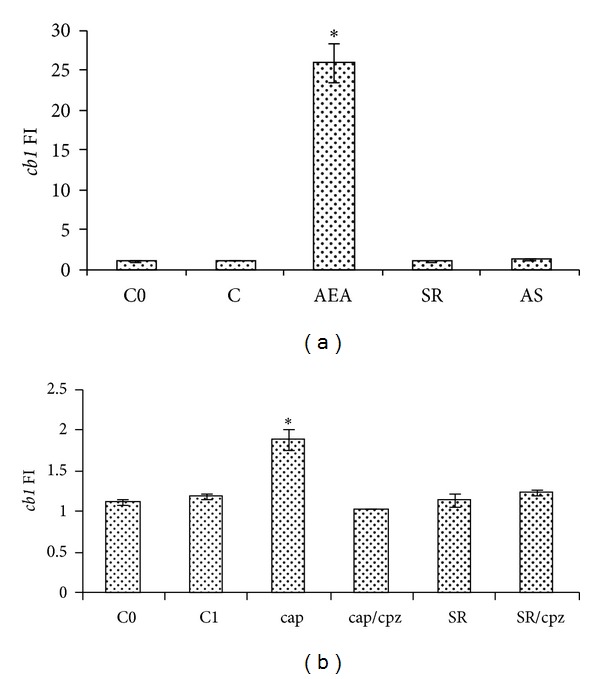
Effects of AEA treatment on *cb1* (a) expression in frog testis collected from June animals (*N* = 5/group) after 1 h of incubation. Incubations have been carried out with AEA 10^−9^ M, SR 10^−8^ M, or both. C0: untreated testis of June; C: control group, testis treated with Krebs-Ringer buffer. Effects of cap treatment on *cb1* (b) expression in frog testis of June after 1 h of incubation. Incubations have been carried out with cap 10^−6^ M, cpz 10^−5^ M, SR 10^−8^ M, or combinations of cap/cpz and SR/cpz. C0: untreated testis of June; C1: control group, testis treated with Krebs-Ringer buffer. The data in graph are the results of RT-PCR analysis; they are reported as fold increase (FI) calculated comparing the expression of *cb1 *to the housekeeping *fp1 *and are representative of three separate experiments at least (*N* = 6). Asterisks indicate statistically significant differences.

As mentioned above, AEA has a dual potentiality thanks to the ability to bind to both CB1 and TRPV1 and so working as an endocannabinoid and an endovanilloid as well. In the context of reproduction, this peculiarity makes AEA a dual regulator of acrosome reaction (AR). In boar sperm, AEA—present in both seminal plasma and uterine fluids—prevents, *via* CB1, premature capacitation and inhibits AR [43]. By contrast, a few hours later, when sperm have reached the oviduct, this inhibitory brake becomes less stringent, since AEA concentration progressively reduces. At this time, AEA works as endovanilloid activating TRPV1 [43]. Such an activation prevents spontaneous AR, an uncontrolled phenomenon of exocytosis that leads quickly to cell death [70]. Besides functions related to fertilizing ability due to intracellular AEA signalling, few and contradictory studies have analyzed the effects of CAP, the agonist of TRPV1, in male germ cell progression. In the past, CAP, acting as specific neurotoxin that irreversibly caused degeneration of sensory C fibres of the peripheral nerves, was investigated for its ability to affect testicular descent [71]. However, CAP has been reported to adversely affects the survival of rat spermatogonial cell lines expressing TRPV1 [72], whereas a protective role against heat stress has been suggested for TRPV1 [73]. Conversely in mouse, a diet containing 0.02% CAP enhances testicular cell proliferation and affects the release of both testosterone and ghrelin, the latter being an acylated polypeptide hormone mainly secreted by the endocrine cells of the stomach [74]. Interestingly, in mammals, TRPV1 is expressed in Sertoli cells [75] and germ cells, with high levels of both mRNA and protein detected from spermatocytes to spermatids stages [55]. At present, none has investigated a possible role of endovanilloids in GnRH signalling, either at central level or at testicular level. Once again a simple animal model as *R. esculenta* has shed light on such a mechanism. In parallel to AEA treatment of frog testis, in June, *in vitro* stimulation with CAP has been carried out. Interestingly, the effects observed upon GnRH system have been opposite to those of AEA. In particular, CAP increases *GnRH-1 *and decreases *GnRH-2* (Figure 3); then, it decreases *GnRH-R1* and increases *GnRH-R2*, with no effect on *GnRH-R3* (Figure 4). These effects have been completely counteracted by capsazepine (CPZ), a competitive TRPV1 antagonist [76]. No effects have been observed after SR141716A (SR), a CB1 antagonist, alone or in combination with CPZ. Interestingly, CAP affects *cb1* expression as well (Figure 5) suggesting a possible overlapping between the eCB and the endovanilloid system. 

## 5. Closing Remarks

The eCS field is an important example of the kinds of inputs that studies of comparative endocrinology can give to our knowledge. The contribution of lower vertebrate animal models in reproduction research is very strong not only because they make easy the investigation of mechanisms regulating mammalian reproductive physiology but also because they allow to understanding on how these mechanisms have evolved.

The frog *R. esculenta* has been a suitable model for a complete characterization of the eCS. Thanks to its feature as seasonal breeder, *GnRH* and *cb1* expression profiles have been compared indicating the existence of a physiological reverse relationship between the two systems. More interestingly what happens in brain not always can be confirmed in testis; in fact, a different regulation by AEA of the GnRH system has emerged in frog brain and testis. In addition, a novel role can be ascribed to endovanilloids as new regulators of the GnRH system in testis. Furthermore, it is reasonable that eCBs and endovanilloids might work as two different faces of the same medal since an opposite regulation of each component of the GnRH system by these molecules has been described.
